# Floral Quality Characterization in Olive Progenies from Reciprocal Crosses

**DOI:** 10.3390/plants11101285

**Published:** 2022-05-11

**Authors:** Hava F. Rapoport, Inmaculada Moreno-Alías, Miguel Ángel de la Rosa-Peinazo, Amina Frija, Raúl de la Rosa, Lorenzo León

**Affiliations:** 1 Instituto de Agricultura Sostenible, Consejo Superior de Investigaciones Científicas, Avda. Menéndez Pidal, Campus Alameda del Obispo, s/n, 14004 Córdoba, Spain; inmaculada.moreno.a@gmail.com (I.M.-A.); mdelarosa@panuveg.es (M.Á.d.l.R.-P.); amina.frija.tn@gmail.com (A.F.); 2 Departamento de Agronomía, Universidad de Córdoba, 14014 Córdoba, Spain; 3 IFAPA Centro Alameda del Obispo, Junta de Andalucía, Avda. Menéndez Pidal, s/n, 14080 Córdoba, Spain; lorenzo.leon@juntadeandalucia.es

**Keywords:** *Olea europea* L., breeding, inflorescence, ovule, ovary, mesocarp, ‘Arbequina’, ‘Picual’, perfect flower, staminate flower

## Abstract

Despite the importance of flowering for fruit formation, it has been considered very little in breeding programs involving fruit species, including olives. We evaluated the principal morphological flower-quality components in the olive cultivars, ‘Arbequina’ and ‘Picual’, and in the progenies of their crosses. Wide ranges of variation were obtained for all the inflorescence traits and ovary tissue sizes. An analysis of variance indicated that the residual error was the main contributor to the inflorescence traits, except for the number of perfect flowers, underlining the need to evaluate adequate numbers of inflorescences for accurate measurements of these traits. However, the high repeatability obtained for the inflorescence traits suggests that simple evaluation procedures could be accurate enough for genotype characterization. The average values for ‘Arbequina’ were in the upper range for all the traits; the opposite occurred for ‘Picual’, and the values for most of the progenies were intermediate. No significant differences between the maternal and paternal effect on inheritance were found. Some interesting transgressive segregants showed a higher flower number, greater ovary and mesocarp size, or percentage of ovaries with all four fully developed ovules. The correlations among the parameters may have reflected a relatively consistent distribution of the ovaries’ structural components and a close relationship between the ovaries and their mesocarp growth.

## 1. Introduction

Flowering is the critical first step in fruit formation; however, it has been evaluated very rarely in breeding programs for olive or other fruit tree species [1]. Some of the reasons for this are the long juvenile periods in these species, a yearly or biennial cycle of events affecting flowering, large numbers of flowers with variable and physiologically compensating parameters, and time-consuming procedures for the evaluation of many flower characteristics. Among the few breeding studies that consider flower development in tree crops, McNeilage [2] found that the tendency to develop bisexual flowers in kiwifruit progenies is inherited. Somewhat more numerous are the studies regarding the genetic variability and inheritance of floral developmental timing, such as in almonds [3], the apricot flowering period [4], and the kiwifruit flowering time [5,6]. Both maternal and paternal effects on the apple blooming period were found by Soltész [7], with the blooming time determined by the paternal parent and the flower initiation by the maternal parent.

Fruit-tree flower quality encompasses the multiple aspects of floral structure and physiology that potentially affect fruit number and/or size [8]. In olive trees, in addition to the inflorescence and flower number, the fruit number is potentially limited by two frequent morphological failures: pistil abortion and incomplete ovule development [9]. In the andromonoecious sexual system of olive trees, pistil abortion produces higher numbers of staminate (imperfect) flowers at the expense of hermaphrodite (perfect) flowers [10,11]. Only the hermaphrodite flowers contain ovaries, so their presence is fundamental in determining the fruit number [12,13]. Pistil abortion is influenced by environmental and nutritional conditions [14,15,16,17]. Furthermore, as also occurs in other species in the plant kingdom [18], this trait has a clearly recognizable genetic component in olive trees [9,11,19,20,21,22].

Once a hermaphrodite flower with a well-formed ovary is present, the fruit number is determined by its capacity for successful fertilization. In olive trees, fertilization can be limited by incomplete or anomalous ovule development, in which the embryonic sac fails to fully differentiate [15,23]. As with pistil abortion, both environmental and genetic factors influence ovule development [9]. Incomplete ovule development may be associated with pistil abortion, as suggested by the presence of ovules without embryonic sacs observed by Reale et al. [11] in staminate flowers. It remains to be determined, however, whether both tendencies may simply respond to common resource limitations, or if they are genetically linked to each other [9].

A third relevant morphological component of olive flower quality, which potentially influences fruit size rather than number, is ovary size. Among a range of olive cultivars, Rosati et al. [24] found that fruit weight was related to ovary weight, and Rosati et al. [25] observed that ovary mesocarp and endocarp tissue sizes determine future tissue growth and partitioning in the fruit.

Even though a strong genetic (cultivar) influence has been reported for the above- described floral morphogenetic components, the inheritance of these traits has been seldom studied in the progenies of olive crosses. In the new olive cultivar, ‘Sikitita’ (known in USA as ‘Chiquitita’) [26], flower number per inflorescence was similar to its male parent ‘Arbequina’, whereas perfect flower production was intermediate in the two parent cultivars ‘Arbequina’ and ‘Picual’ [27]. Similarly, the ovule development quality was similar to its male parent, ‘Arbequina’, and significantly different from its female parent, ‘Picual’ [27].

In this study, we evaluated the principal morphological components of flower quality in the olive cultivars ‘Arbequina’ and ‘Picual’, and in the progenies of their reciprocal crosses, in order to observe indications of their inheritance and determine whether any particular maternal or paternal effect could be identified. In relation to the flower-quality factors determining the final fruit number, we examined the inflorescence flower number, pistil abortion, and incomplete ovule development. In relation to the determination of the fruit size, we evaluated the ovary and ovary tissue sizes, and tested their correlation with the mature fruit tissues.

## 2. Results and Discussion

### 2.1. Variability in Inflorescence and Ovary Traits

Wide ranges of variation were obtained for all the inflorescence traits and ovary tissue sizes, with the greatest coefficients of variation found in the numbers and percentages of perfect flowers (Table 1). The values obtained for both the inflorescence and ovary development parameters were similar to those observed in previous studies of the flower quality of ‘Arbequina’ and/or ‘Picual’, the two parental cultivars [19,27], as were the measurements of the ovary sizes [16,25].

**Figure 1:**
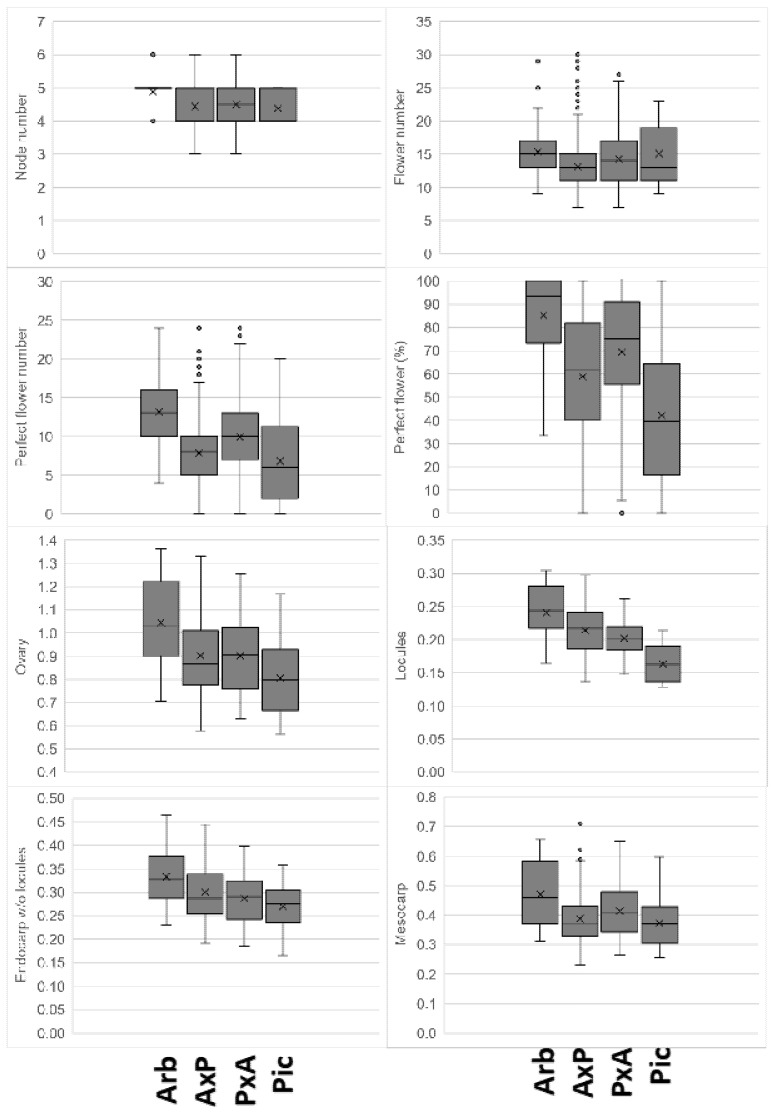
Flower quality parameter boxplots for ‘Arbequina’ (Arb) and ‘Picual’ (Pic) and eight seedlings of each of their reciprocal crosses (A × P and P × A). Horizontal box lines indicate the 25th, 50th, and 75th percentiles; whiskers indicate range, excluding outliers (points).

The ‘Arbequina’ consistently showed higher values than the ‘Picual’ for all the inflorescence and ovary parameters (Figure 1), as was previously reported for the new cultivar ‘Sikitita’ [27]. Regarding the reciprocal crosses, no differences were observed between ‘Arbequina’ × ‘Picual’ vs. ‘Picual’ × ‘Arbequina’, so any potential maternal or paternal effect on the inheritance of these characters can be excluded. A wide experimental variability was observed for all the traits. Thus, the range of the 50% of values closest to the median, indicated by the height of the data box (Figure 1), was often similar or greater for the parent genotypes than for their progenies, even though the progeny data corresponded to eight individuals per cross, and the parent data to one plant per genotype.

The variance components showed different contributions of the sources of variation for the different evaluated traits (Table 2). The residual error σ²ε was the main contributor to the inflorescence traits, except for the perfect flower number. This observation underlines the need to evaluate an adequate number of inflorescences for accurate the measurement of these traits. On the other hand, the σ²G contribution was higher than that of the σ²GY, while the σ²Y was particularly high for the perfect flower number. For the ovary tissue sizes, the σ²G and σ²Y contributed, together, to around 70% of the variance, with a much lower contribution from the σ²ε and the σ²GY. It should be noted that the variance among the trees within a genotype was not considered in our study, although a negligible contribution of this factor was reported previously [27].

These results provided repeatability estimates from 0.63 to 0.76 for the inflorescence traits, and slightly higher values (0.82–0.85) for the ovary tissue sizes (Table 2). The high repeatability obtained for these characters suggests that simple evaluation procedures could be accurate enough to characterize the genotypes for these traits, which is particularly useful for screening new genotypes in breeding programs. Similar high-repeatability values were previously reported from the evaluation of olive progenies for related traits, such as ripening date and fruit size [28].

**Figure 2:**
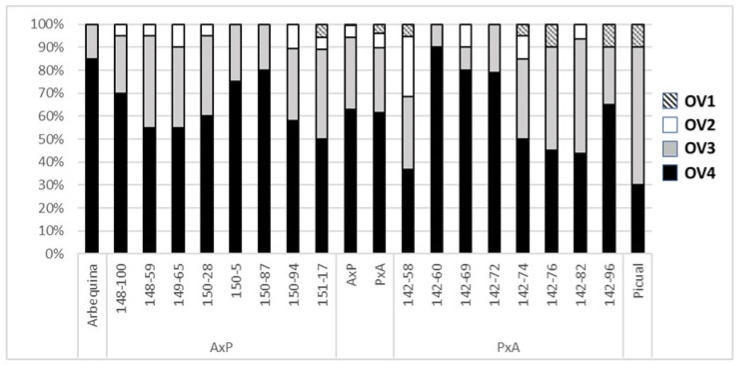
Ovary rating distribution (percentage) for each genotype, according to the number of normal, fully developed ovules of the four present in each ovary. Ovary categories are from OV1 (one fully developed ovule) to OV4 (four fully developed ovules). Distribution is shown for parent cultivars, ‘Arbequina’ and ‘Picual’, each individual progeny genotype, and means for all progenies of both crosses.

Significant differences (Table 2) were obtained for all the components (the genotype, the year, and their interaction) of the statistical model for all the evaluated traits. Significant genotypic, environmental, and interaction effects were previously reported for several inflorescence quality parameters, as well as phenological traits [19]. The comparison of means of the interaction term (Table 3) showed, as a general trend, average values for ‘Arbequina’ in the upper range for all the traits and the opposite for ‘Picual’, as was previously mentioned regarding the general variability evaluation in Figure 2. It should be noted, however, that some transgressive segregants were observed in both the inflorescence traits and the ovary tissue sizes (Table 3). Thus, for instance, the highest values for the flower number and ovary size were found for genotype 142–82 in 2014 and 142–96 in 2013, respectively.

All the parameters evaluated showed higher values in 2013 compared to 2014, especially the number and percentage of perfect flowers (Table 3). This could have been due to the higher temperature and lower rainfall in 2014, particularly from March to May (Table 4), when olive flowers develop.

### 2.2. Relations among Studied Parameters

The similar pattern of variability inferred for both inflorescence and ovary parameters in Figure 1 suggests a potentially high correlation among them. Thus, a significant correlation was observed for the number and percentage of perfect flowers (Table 5). This might have been expected, given the similar total flower number among all the groups in our work, although it might have varied if the parent genotypes had differed in inflorescence flower number. Similarly, a high correlation was obtained among the ovary tissue size parameters, particularly for the pair ovary and mesocarp size (Table 5). These results may reflect the relatively consistent distribution of the ovaries’ structural components and a close relationship between the ovaries and their mesocarp growth.

No significant correlation, however, was found between the ovary tissue size parameters and the corresponding mature fruit tissues (fruit, pulp, and pit transverse area) (data not shown). By contrast, Rosati et al. [24] found a strong correlation between the ovaries and the fruit dry weight among olive cultivars with a wide range of fruit sizes. This difference in results may be related to the experimental data range, as Rosati et al [24].’s genotypes included a 6-to-7-fold range in both fruit and ovary sizes, while our progeny genotypes ranged less than 2-fold for both ovary and fruit transverse area. The environmental conditions during fruit growth were identical among the genotypes, as all the plants grew side by side under the same conditions. The lack of ovary–fruit correlations for the evaluated genotypes might have been due to the greater importance of the ovary/fruit cell number and cell division capacity compared with the ovary and ovary cell size, which have been reported in olives [25,29,30], and other fruit species, such as plums [31], apples [32], and peaches [33]. Nevertheless, our current progeny study did not include ovary cell observations. The relationship between olive ovary tissue sizes and final fruit sizes deserves further investigation, but our observations suggest that, at this time, it is more pragmatic for breeders to select for fruit size by directly using fruit rather than ovary measurements.

### 2.3. Ovule Development

The ovary rating, based on the number of fully developed ovules of the four present in each ovary, also showed variability among progeny genotypes (Figure 2). The percentages of the ovaries with each rating (number of fully developed ovules) were almost identical in the two evaluated years (X^2^ = 0.09, *p* = 0.9929), and no significant differences obtained among the cross combinations (X^2^ = 4.24, *p* = 0.2368). Significant differences were found, however, among the genotypes (X^2^ = 85.06, *p* = 0.0019, Figure 2). Again, the average values for the ‘Arbequina’ showed a higher percentage of ovaries, with four fully developed ovules, than the ‘Picual’ (85% vs. 30% respectively). In fact, among both progenies and parents, the ‘Picual’ showed the lowest percentage of ovaries, with four fully developed ovules. The distribution among the genotypes was more similar when using the combined percentage of ovaries with three or four fully developed ovules, the values considered to represent good possibilities for fertilization [23,27], which accounted for 85–100% in all the genotypes, except for 142–58.

## 3. Materials and Methods

### 3.1. Plant Material and Growing Conditions

The plants under study belong to the olive breeding program of the IFAPA (Andalusian Institute of Agricultural and Fisheries Research and Training) at the Alameda del Obispo Center in Córdoba, Spain. Five-year-old adult seedling trees from reciprocal crosses of cultivars ‘Arbequina’ and ‘Picual’ (eight genotypes A × P, maternal parent ‘Arbequina’, paternal parent ‘Picual’; and eight genotypes P × A) were grown together with vegetatively cloned parent cultivars of the same age. Only one plant per genotype was evaluated per parent cultivar in order to be comparable with the single-plant genotypes of the crosses. Trees were drip-irrigated with 1500 m^3^ per ha and year. Training was performed to form a canopy at a 1-meter height, which was then allowed to develop freely to maximize productivity. Phytosanitary treatments were performed twice a year to prevent the appearance of peacock spot. Nitrogen fertilization was applied to promote plant growth. Daily temperature and rainfall data were obtained from the Alameda del Obispo weather station of the Andalusian Agroclimatic Information Network. This station is located less than 200 m from the sampled olive trees.

### 3.2. Inflorescence and Flower Sampling and EVALUATION

Inflorescences were sampled and evaluated in two successive years (2013 and 2014), following the procedures in [27]. During full bloom, twenty-five inflorescences were collected around the canopy of each of the selected olive trees and fixed in FAE (formalin: acetic acid: 50% ethanol = 2:1:17 *v*/*v*/*v*). A mixture of open and closed flowers in the sampled inflorescences guaranteed the presence of recently opened flowers containing mature ovaries just prior to fertilization. Following transfer to 70% ethanol and step-wise rehydration to 30% ethanol, the number of nodes, number of flowers, and number and percentage of perfect (hermaphrodite) flowers were determined.

### 3.3. Ovule Development and Ovary Tissue Size

Ten pistils per genotype and year were selected from perfect flowers interpreted as having recently opened on the sampled inflorescences, using a maximum of two pistils per inflorescence. The pistils were processed in Histosec^®^ embedding paraffin with a melting point 56–58 °C (Merck, Darmstadt, Germany), according to standard paraffin procedures [34]; the ovaries were sectioned transversely at 12 μm and stained with toluidine blue (0.05%) [35].

**Figure 3:**
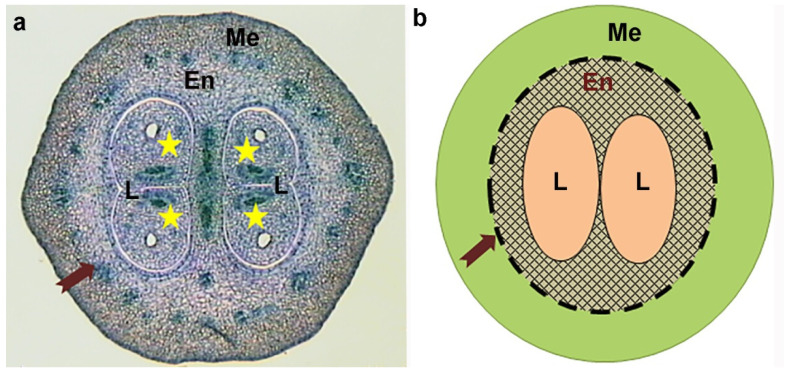
Central transverse section of olive ovary at bloom (**a**) and diagram showing the measured structures (**b**). The mesocarp (Me) and endocarp (En) are separated by a ring of vascular bundles (arrow); the two locules (L) each contain two ovules (star).

The ovules of the ten processed pistils were observed in successive transverse ovary sections by optical microscope (Leica DMRB-FHC, Leica Microsystems, Heerbrugg, Switzerland). Each ovary was rated 0–4, according to the number of fully developed ovules (containing totally differentiated embryo sacs) found among the total of four ovules normally present in the olive [9,27] (Figure 3a). The percentages of ovaries with each rating (number of fully developed ovules) were calculated for each genotype and year.

Ovary and ovary tissue sizes were determined in central transverse sections of the ovaries previously used for ovule analysis. Only ovaries with three or four developed ovules were used for the measurements, to eliminate any link with poor or anomalous ovule development and reduced ovary growth [23]. In order to obtain ten ovaries that fulfilled the requirement for enough developed ovules, additional pistils were used from the same initial inflorescence sample when necessary. Images were captured with a digital camera (Leica DFC450C) and the tissues were measured using an image analysis system (LAS v.4, Leica, Cambridge, UK) connected to the microscope indicated above. Ovary, endocarp (including locules), and locule areas were measured; mesocarp area and endocarp (without locule area) were calculated by subtraction (Figure 3).

### 3.4. Mature Fruit Tissues

In the second year (2014) of the experiment, mature green fruits were also sampled, fixed, and later rehydrated for measurement, as described above for the inflorescences. For ten fruits per tree, the central transverse diameters of fruit and pit were measured and the relations of fruit, pulp, and pit to ovary, ovary mesocarp, and ovary endocarp size were evaluated.

### 3.5. Data Analysis

Descriptive statistics of the different traits were obtained for the whole dataset. ANOVA was applied according to the following statistical model: Pijk = µ+Gi + Yj + (G × Y)ij + εijk, where Pijk is the phenotypic value of the k sample of the i genotype in the j year, µ is the overall mean of the progeny, Gi is a random effect contributed by the i genotype, Yj is a random effect of the j year, (G × Y)ij is the interaction between the i genotype and the j year, and εijkl is the random residual error effect for the k measured samples. ANOVA provided the variance among genotypes (σ²G), among years (σ²Y), associated with the genotype × year interaction (σ²GY), and residual error effect for the measured samples (σ²ε). From this model, repeatability was estimated as r = σ²G/ (σ²G + σ²GY/y + σ²ε/ys), where y is the number of years (2) and s the number of samples (25 for inflorescence traits and 10 for ovary tissue size). Comparison of means was carried out according to Tukey’s HSD test at *p* = 0.05. The correlations between ovary and fruit tissues were tested by determining the Pearson coefficient (*p* = 0.05).

## 4. Conclusions

The evaluation of genotypes from the reciprocal crosses between ‘Arbequina’ and ‘Picual’ showed mostly intermediate values for the studied flower quality traits in the progenies, with no significant differences between the maternal and the paternal effect on the inheritance. Furthermore, some interesting transgressive segregants showed higher flower numbers, greater ovary and mesocarp tissue sizes, or higher percentages of ovaries with four fully developed ovules. It is remarkable that among the descendants of the two cultivars used as parents, ‘Arbequina’ and ‘Picual’, only a few genotypes should be potentially discarded on the basis of very low values in these traits. Despite the importance of flower quality for final fruit production, its evaluation is time-consuming, particularly when histological procedures are involved, and requires careful sampling and large sample numbers. The large differences between the two parents for the evaluated flower quality parameters produced a high level of variability among the descendants. This high variability allows us to conclude that the flower-quality parameters that influence olive fruit number, such as flower number, perfect flower number, and ovule development, appear to be more pertinent for breeding programs due to the high genetic influence, whereas the link between ovary quality and fruit quality is still unclear. These assumptions, however, should be further confirmed with studies in other progenies involving different cultivars as genitors.

## Figures and Tables

**Table 1:** Overall variability in inflorescence and ovary parameters for all genotypes (parents and progenies) in the two years of study.

	Min	Max	Mean	SD	CV (%)
Inflorescence traits					
Node number	3	6	4.49	0.59	13.23
Flower number	7	30	13.87	3.89	28.04
Perfect flower number	0	24	9.02	4.71	52.19
Perfect flower (%)	0	100	64.14	27.49	42.86
Tissue size (mm^2^)					
Ovary	0.52	1.41	0.91	0.18	19.22
Locules	0.13	0.30	0.21	0.03	16.07
Endocarp w/o locules	0.16	0.52	0.30	0.06	21.13
Mesocarp	0.20	0.71	0.40	0.09	23.08

**Table 2:** Variance components in ANOVA for the different inflorescence and ovary parameters in the sixteen seedlings and two parents (‘Picual’ and‘Arbequina’). Significant differences at *p* < 0.01 were obtained for all factors (genotype, year, and genotype × year) and traits.

	**Inflorescence Traits**							
	**Node Number**		**Flower Number**		**Perfect Flower Number**		**Perfect Flower (%)**	
	**Variance**	**%**	**Variance**	**%**	**Variance**	**%**	**Variance**	**%**
Genotype (G)	0.0376	9.6	3.1	17.5	3.6	12.6	197.7	22.4
Year (Y)	0.0712	18.2	4.6	26.1	12.0	42.3	227.9	25.8
G × Y	0.0275	7.0	1.9	10.8	3.8	13.3	113.3	12.8
Error	0.2546	65.1	8.0	45.6	9.1	31.9	344.2	39.0
Repeatability	0.67		0.74		0.63		0.76	
	**Tissue Size**							
	**Ovary**		**Locules**		**Endocarp w/o Locules**		**Mesocarp**	
	**Variance**	**%**	**Variance**	**%**	**Variance**	**%**	**Variance**	**%**
Genotype (G)	0.0123	30.5	0.0006	39.1	0.0018	36.7	0.0032	28.1
Year (Y)	0.0168	41.8	0.0005	33.2	0.0015	30.3	0.0047	42.3
G × Y	0.0042	10.4	0.0002	12.2	0.0006	12.0	0.0011	9.9
Error	0.0069	17.2	0.0002	15.5	0.0010	21.0	0.0022	19.7
Repeatability	0.83		0.85		0.84		0.82	

**Table 3:** Average values by genotype and year for the evaluated inflorescence and ovary parameters.

**Cross**	**Genotype**	**Inflorescence Traits**							
		**Node Number**		**Flower Number**		**Perfect Flower Number**		**Perfect Flower (%)**	
		**2013**	**2014**	**2013**	**2014**	**2013**	**2014**	**2013**	**2014**
	Arbequina	5.12	4.64	17.1	13.6	16.24	10.08	96.0	74.5
A × P	148–100	4.56	4.12	13.8	11.2	6.84	4.88	49.6	43.1
	148–59	4.56	4.52	15.1	13.0	12.84	9.4	84.8	74.7
	149–65	4.92	4.4	17.6	12.1	15.96	5.64	90.6	47.3
	150–28	4.72	4	15.0	11.3	11.56	6.4	77.0	59.2
	150–5	4.72	3.88	15.7	11.0	8.08	2.8	53.1	24.5
	150–87	4.28	4.32	10.8	11.6	9.76	6.76	90.0	60.2
	150–94	5.36	4.68	18.6	11.8	8.48	4.56	46.0	35.3
	151–17	4.12	3.88	10.6	11.0	4.52	6.96	43.4	63.3
P × A	142–58	4.32	4.32	15.7	13.0	14.48	7.36	91.7	56.9
	142–60	4.88	4.52	16.6	12.3	14.04	5.2	85.5	41.9
	142–69	4.64	4.4	14.6	10.1	9.96	3.32	68.5	33.2
	142–72	4.72	4.04	14.2	9.8	12.52	6.2	87.9	62.2
	142–74	4.52	4.2	13.3	11.3	12.32	10.08	92.7	90.3
	142–76	4.92	4.16	14.6	10.7	12.24	7.16	84.3	66.3
	142–82	4.64	4.56	19.0	19.1	11.84	8.04	62.6	42.1
	142–96	4.68	4.44	17.3	16.4	14.68	9.76	84.3	61.2
	Picual	4.52	4.24	17.6	12.6	10.56	3.12	61.4	23.1
	Tukey HSD 95%	0.5474		3.0717		3.264		20.106	
		**Tissue Size (Central Transverse Area, mm^2^)**							
**Cross**	**Genotype**	**Ovary**		**Locules**		**Endocarp w/o Locules**		**Mesocarp**	
		**2013**	**2014**	**2013**	**2014**	**2013**	**2014**	**2013**	**2014**
	Arbequina	1.218	0.869	0.275	0.206	0.380	0.286	0.564	0.378
A × P	148–100	0.858	0.766	0.189	0.168	0.307	0.271	0.361	0.328
	148–59	1.125	0.808	0.229	0.183	0.353	0.274	0.543	0.351
	149–65	0.826	0.657	0.219	0.181	0.250	0.211	0.357	0.265
	150–28	1.168	1.037	0.259	0.253	0.400	0.347	0.509	0.438
	150–5	0.846	0.610	0.237	0.172	0.260	0.204	0.350	0.233
	150–87	0.957	0.814	0.246	0.214	0.316	0.269	0.395	0.331
	150–94	1.020	0.914	0.198	0.201	0.373	0.308	0.449	0.406
	151–17	0.845	0.967	0.209	0.210	0.262	0.349	0.374	0.408
P × A	142–58	1.016	0.796	0.220	0.195	0.311	0.253	0.485	0.348
	142–60	0.915	0.709	0.198	0.168	0.307	0.226	0.409	0.316
	142–69	1.038	0.846	0.234	0.201	0.323	0.264	0.480	0.381
	142–72	0.937	0.795	0.224	0.200	0.270	0.230	0.444	0.365
	142–74	0.969	0.708	0.230	0.175	0.306	0.219	0.433	0.314
	142–76	1.101	0.914	0.208	0.187	0.355	0.299	0.538	0.428
	142–82	1.151	0.927	0.258	0.234	0.417	0.336	0.477	0.357
	142–96	1.260	1.066	0.267	0.241	0.413	0.346	0.580	0.480
	Picual	0.935	0.675	0.184	0.142	0.310	0.229	0.441	0.304
	Tukey HSD 95%	0.1921		0.0343		0.0747		0.1089	

**Table 4:** Average monthly values for daily average, maximum and minimum temperature, and cumulative rainfall for January to May in the two years (2013 and 2014) of the present study.

Year	Month	Temperature (°C)			Cumulative Rainfall (mm)
		Average	Max	Min	
2013	January	8.66	14.51	4.24	61.80
	February	8.85	14.92	3.33	87.40
	March	12.05	16.78	8.28	266.40
	April	15.69	22.33	9.69	51.20
	May	18.11	25.31	10.75	12.20
2014	January	8.19	14.73	3.40	103.60
	February	10.15	14.96	5.34	108.20
	March	12.70	20.01	6.34	30.60
	April	17.35	24.59	10.66	52.60
	May	20.71	28.70	12.07	9.40

**Table 5:** Pearson correlation coefficient for inflorescence and ovary parameters evaluated in two consecutive years in the sixteen seedlings and two parents (‘Picual’ and ‘Arbequina’). Significant correlations at *p* < 0.05 are indicated in bold.

		Node Number	Flower Number	Perfect Flower Number	Perfect Flower (%)	Ovary Size	Endocarp w/o Locules Size	Locules Size
Flower number	2013	**0.67**						
	2014	**0.53**						
Perfect flower number	2013	0.36	0.49					
	2014	0.29	0.45					
Perfect flower (%)	2013	0.06	−0.01	**0.85**				
	2014	0.00	−0.01	**0.88**				
Ovary size	2013	0.27	0.35	**0.47**	0.35			
	2014	0.11	0.33	0.42	0.30			
Endocarp w/o locules size	2013	0.32	0.44	0.28	0.09	**0.92**		
	2014	0.09	0.41	0.37	0.20	**0.95**		
Locules size	2013	0.04	0.08	0.40	0.41	**0.68**	**0.52**	
	2014	0.08	0.39	0.42	0.28	**0.85**	**0.78**	
Mesocarp size	2013	0.25	0.31	**0.53**	0.43	**0.95**	**0.78**	**0.51**
	2014	0.12	0.18	0.41	0.35	**0.95**	**0.84**	**0.70**

## Data Availability

Not applicable.

## References

[1] Badenes M.L., Byrne D.H. (2012). Fruit Breeding.

[2] McNeilage M.A. (1991). Gender Variation in Actinidia Deliciosa, the Kiwifruit. Sex. Plant Reprod..

[3] Sánchez-Pérez R., Dicenta F., Martínez-Gómez P. (2012). Inheritance of Chilling and Heat Requirements for Flowering in Almond and QTL Analysis. Tree Genet. Genomes.

[4] Campoy J.A., Ruiz D., Egea J., Rees D.J.G., Celton J.M., Martínez-Gómez P. (2011). Inheritance of Flowering Time in Apricot (*Prunus Armeniaca* L.) and Analysis of Linked Quantitative Trait Loci (QTLs) Using Simple Sequence Repeat (SSR) Markers. Plant Mol. Biol. Rep..

[5] Cheng C.H., Seal A.G., Murphy S.J., Lowe R.G. (2006). Variability and Inheritance of Flowering Time and Duration in Actinidia Chinensis (*Kiwifruit*). Euphytica.

[6] Ruiz D., Egea J. (2007). Ovule Development at Anthesis in Apricot (Prunus Armeniaca) Varieties in a Mediterranean Climate. Ann. Appl. Biol..

[7] Soltész M. (2000). Inheritance of the Characters Related to Reproductive Processes in Apple Varieties: Flower Initiation, Blooming, Fertility Conditions. Acta Hortic..

[8] Williams R.R. (1965). The Effect of Summer Nitrogen Applications on The Quality of Apple Blossom. J. Hortic. Sci..

[9] Moreno-Alías I., Rapoport H.F., Martins P.C. (2012). Morphological Limitations in Floral Development among Olive Tree Cultivars. Acta Hortic..

[10] Cuevas J., Polito V.S. (2004). The Role of Staminate Flowers in the Breeding System of Olea europaea (Oleaceae): An Andromonoecious, Wind-Pollinated Taxon. Ann. Bot..

[11] Reale L., Sgromo C., Bonofiglio T., Orlandi F., Formaciari M., Ferranti F., Romano B. (2006). Reproductive Biology of Olive (*Olea Europea* L.) DOP Umbria Cultivars. Sex. Plant Reprod..

[12] Lavee S., Rallo L., Rapoport H.F., Troncoso A. (1996). The Floral Biology of the Olive: Effect of Flower Number, Type and Distribution on Fruitset. Sci. Hortic..

[13] Rallo L., Fernandez-Escobar R. (1985). Influence of Cultivar and Flower Thinning within the Inflorescence on Competition among Olive Fruit. J. Am. Soc. Hortic. Sci..

[14] Perica S., Brown P.H., Connell J.H., Nyomora A.M.S., Dordas C., Hu H., Stangoulis J. (2001). Foliar Boron Application Improves Flower Fertility and Fruit Set of Olive. HortScience.

[15] Rallo L., Martin G.C., Lavee S. (1981). Relationship between Abnormal Embryo Sac Development and Fruitfulness in Olive. J. Am. Soc. Hortic. Sci..

[16] Rapoport H.F., Hammami S.B.M., Martins P., Pérez-Priego O., Orgaz F. (2012). Influence of Water Deficits at Different Times during Olive Tree Inflorescence and Flower Development. Environ. Exp. Bot..

[17] Uriu K. (1960). Periods of Pistil Abortion in the Development of the Olive Flower. J. Am. Soc. Hortic. Sci..

[18] Boualem A., Lemhemdi A., Sari M.-A., Pignoly S., Troadec C., Choucha F.A., Solmaz I., Sari N., Dogimont C., Bendahmane A. (2016). The Andromonoecious Sex Determination Gene Predates the Separation of Cucumis and Citrullus Genera. PLoS ONE.

[19] Navas-Lopez J.F., León L., Rapoport H.F., Moreno-Alías I., Lorite I.J., de la Rosa R. (2019). Genotype, Environment and Their Interaction Effects on Olive Tree Flowering Phenology and Flower Quality. Euphytica.

[20] Koubouris G.C., Metzidakis I.T., Vasilakakis M.D. (2010). Phenological, Morphological and Functional Indicators of Genetic Variability and Their Implication in the Sexual Reproductive System of *Olea europaea* L. (Oleaceae). Sci. Hortic..

[21] Vuletin Selak G., Perica S., Goreta Ban S., Bucan L., Poljak M. (2012). Flower Sterility and the Germination Ability of Pollen as Genetic Traits of Seven Olive (*Olea europaea* L.) Cultivars Grown in Croatia. J. Hortic. Sci. Biotechnol..

[22] Rosati A., Caporali S., Paoletti A., Famiani F. (2011). Pistil Abortion Is Related to Ovary Mass in Olive (*Olea europaea* L.). Sci. Hortic..

[23] Martins P.C., Cordeiro A.M., Rapoport H.F. (2006). Flower Quality in Orchards of Olive, Olea europaea L., Cv. Morisca. Adv. Hortic. Sci..

[24] Rosati A., Zipancic M., Caporali S., Padula G. (2009). Fruit Weight Is Related to Ovary Weight in Olive (*Olea europaea* L.). Sci. Hortic..

[25] Rosati A., Caporali S., Hammami S.B.M.M., Moreno-Alias I., Paoletti A., Rapoport H.F. (2012). Tissue Size and Cell Number in the Olive (*Olea europaea*) Ovary Determine Tissue Growth and Partitioning in the Fruit. Funct. Plant Biol..

[26] Rallo L., Barranco D., de la Rosa R., León L. (2008). ‘Chiquitita’ Olive. HortScience.

[27] Moreno-Alias I., De la Rosa R., Rapoport H.F. (2013). Floral Quality Components of a New Olive Cultivar and Its Parents. Sci. Hortic..

[28] León L., Rallo L., Del Río C., Martín L.M. (2004). Variability and Early Selection on the Seedling Stage for Agronomic Traits in Progenies from Olive Crosses. Plant Breed..

[29] Rapoport H.F., Hammami S.B.M., Rosati A., Gucci R. (2017). Advances in Olive Fruit Cell and Tissue Development. Acta Hortic..

[30] Rosati A., Caporali S., Hammami S.B.M., Moreno-Alías I., Rapoport H. (2020). Fruit Growth and Sink Strength in Olive (Olea europaea) Are Related to Cell Number, Not to Tissue Size. Funct. Plant Biol..

[31] Cerri M., Rosati A., Famiani F., Reale L. (2019). Fruit Size in Different Plum Species (*Genus Prunus* L.) Is Determined by Post-Bloom Developmental Processes and Not by Ovary Characteristics at Anthesis. Sci. Hortic..

[32] Harada T., Kurahashi W., Yanai M., Wakasa Y., Satoh T. (2005). Involvement of Cell Proliferation and Cell Enlargement in Increasing the Fruit Size of Malus Species. Sci. Hortic..

[33] Scorza R., May L.G., Purnell B., Upchurch B. (1991). Differences in Number and Area of Mesocarp Cells between Small- and Large-Fruited Peach Cultivars. J. Am. Soc. Hortic. Sci..

[34] Ruzin S. (1999). Plant Microtechnique and Microscopy.

[35] Sakai W.S. (1973). Simple Method for Differential Staining of Paraffin Embedded Plant Material Using Toluidine Blue O. Stain Technol..

